# Adenoviral Gene Therapy Vectors in Clinical Use—Basic Aspects with a Special Reference to Replication-Competent Adenovirus Formation and Its Impact on Clinical Safety

**DOI:** 10.3390/ijms242216519

**Published:** 2023-11-20

**Authors:** Aleksi J. Leikas, Seppo Ylä-Herttuala, Juha E. K. Hartikainen

**Affiliations:** 1 Heart Center, Kuopio University Hospital, 70200 Kuopio, Finland; seppo.ylaherttuala@uef.fi (S.Y.-H.); juha.hartikainen@pshyvinvointialue.fi (J.E.K.H.); 2 Gene Therapy Unit, Kuopio University Hospital, 70200 Kuopio, Finland; 3 A. I. Virtanen Institute for Molecular Sciences, University of Eastern Finland, 70210 Kuopio, Finland; 4 School of Medicine, Faculty of Health Sciences, University of Eastern Finland, 70210 Kuopio, Finland

**Keywords:** adenovirus, gene therapy, HEK293, oncolytic adenoviruses, replication-competent adenoviruses, vector shedding, VEGF-D

## Abstract

Adenoviral vectors are commonly used in clinical gene therapy. Apart from oncolytic adenoviruses, vector replication is highly undesired as it may pose a safety risk for the treated patient. Thus, careful monitoring for the formation of replication-competent adenoviruses (RCA) during vector manufacturing is required. To render adenoviruses replication deficient, their genomic E1 region is deleted. However, it has been known for a long time that during their propagation, some viruses will regain their replication capability by recombination in production cells, most commonly HEK293. Recently developed RCA assays have revealed that many clinical batches contain more RCA than previously assumed and allowed by regulatory authorities. The clinical significance of the higher RCA content has yet to be thoroughly evaluated. In this review, we summarize the biology of adenovirus vectors, their manufacturing methods, and the origins of RCA formed during HEK293-based vector production. Lastly, we share our experience using minimally RCA-positive serotype 5 adenoviral vectors based on observations from our clinical cardiovascular gene therapy studies.

## 1. Introduction

Gene therapy holds great promise as a method for managing or even curing conditions previously deemed untreatable [1]. Examples of such diseases range from various types of cancer and β-thalassemia to retinal dystrophy, and the U.S. Food and Drug Administration (FDA) has already approved several gene therapy products for clinical use in these indications [2]. In our group, we have investigated adenoviral gene therapy for treating cardiovascular diseases, especially coronary artery disease resistant to conventional treatment options [3].

There are two fundamental categories of gene therapy, utilizing either ex vivo or in vivo transduction. In the former method, donor cells are first harvested from the patient or an individual without the disease. The cells are then transduced using a vector capable of changing their genotype and administered back as modified autologous or allogeneic cell products replacing the deficiently functioning host cells [4]. In in vivo transduction, gene transfer occurs within the patient, and there is a direct interaction between the vectors and the dysfunctional cells of the treated individual [5].

Regardless of the chosen principle, a reliable vector construct is necessary for the efficient transduction of target cells, which is the prerequisite for transgene expression and achieving the desired therapeutic effect. Adenoviruses are one of the most commonly used vectors in clinical trials until 2021 [6], although more recently, the focus of gene therapy vectors has shifted to other viruses, such as adeno-associated viruses and lentiviruses [1]. Adenoviruses offer higher titers and relatively easier large-scale manufacturing than other vectors. They also have several additional benefits, such as good transduction efficacy and retainment of the transgene as an episome without integration into the host genome, making them an attractive platform for transient in vivo gene therapy purposes [7]. Indeed, many recently approved vaccines developed for severe acute respiratory syndrome coronavirus 2 are based on immunization by spike-protein-encoding adenovirus vectors (AdVs) [8].

Despite being generally regarded as having a good safety profile, AdVs are characterized by their high immunogenicity via both innate and adaptive inflammatory responses against the adenoviral capsid structures [9]. In specific contexts, and depending on their magnitude, the host responses to AdVs may lead to fatal consequences, as evidenced by the death of an 18-year-old patient who developed systemic inflammatory response syndrome after receiving a high dose of intravascularly administered adenoviral gene therapy for the treatment of partial ornithine transcarbamylase deficiency [10]. However, it has been noted that there was a problem between the chosen dose and the immune status of the treated patient in this tragic event [11].

A concern that has received much less attention relates to the de novo emergence of replication-competent adenoviruses (RCA) during AdV production [12]. Naturally, RCA itself has been recognized as an essential safety aspect since the dawn of AdV development, and preclinical studies have demonstrated that the presence of RCA is associated with increased inflammation, cytotoxicity, and prolonged vector clearance [13,14,15]. Mirroring the potential safety hazards, the FDA requires the industry to monitor the RCA formation with the upper limit of 1 RCA in 3 × 10^10^ tested viral particles (vp) in the final drug product [16,17]. For our group, this issue returned to the spotlight after analytical methods became sensitive enough to detect RCA in clinical batches that were tested RCA-negative using the previous guideline-recommended assays [18]. This observation resulted in intense correspondence with the local regulatory authorities, eventually approving the use of minimally RCA-positive human adenovirus serotype 5 (HAdV-5)-based gene therapy vectors in our clinical trials.

## 2. Adenoviruses

### 2.1. General Properties

**Figure 1:**
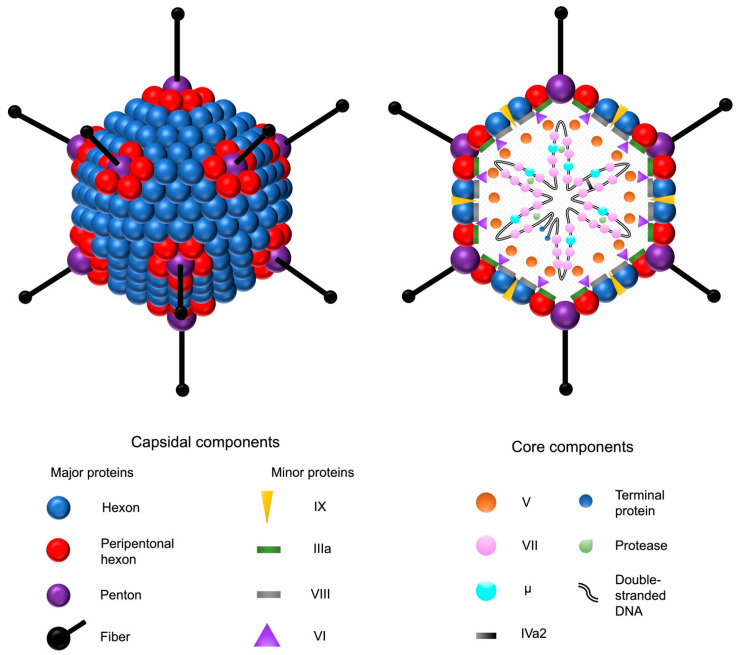
Adenovirus and its structural components. The double-stranded adenovirus DNA is encapsulated by an icosahedral capsid, formed by 252 major capsomers named hexons and pentons. The faces are formed by the hexons, whereas the pentons serve as the bases for fiber shafts that protrude from each vertex and culminate into tip structures called fiber knobs. The fiber proteins are responsible for the interactions between the adenovirus and the host cell receptors. In addition to the major proteins, four minor capsid-associated proteins stabilize the capsid structure. The five core proteins are associated with the adenoviral DNA. Adenovirus protease catalyzes the maturation of the precursor forms of the capsid proteins IIIa, VI, and VIII, as well as the DNA-associated proteins VII, μ, and terminal protein.

Adenoviruses are double-stranded DNA viruses with a non-enveloped, icosahedral capsid of approximately 90 nm in diameter (Figure 1). Their prevalence in the population is high, and they are responsible for common diseases such as rhinitis, gastroenteritis, and conjunctivitis. The family of human adenoviruses is divided into species A–G, which are further classified into many different serotypes [19,20]. The majority of knowledge on adenovirus biology is based on the extensive research of HAdV-2 and HAdV-5, which both belong to subgroup C, the latter being the focus of this review. In addition to humans, different adenoviruses have been isolated from various other vertebrates, such as reptiles, birds, and mammals. No adenovirus identified in other species has been shown to cause clinically significant disease in humans [20].

Adenovirus infection is typically self-resolving and usually causes only mild upper respiratory tract symptoms and fever in immunocompetent individuals. Up to half of all adenovirus infections are asymptomatic or cause only very mild symptoms [19,21]. Adenovirus infections account for approximately 5 to 10% of febrile infections in children and neonates, but their frequency is significantly lower in adults [21]. The incubation time for adenovirus infection is approximately six days [22]. Antibodies against HAdV-5 can be measured in nearly 85% of the population [23].

The most critical risk groups for adenovirus infections are neonates and immunocompromised individuals, in which the adenoviruses may cause severe pneumonia and diseases affecting organ systems other than the respiratory tract, depending on the level of immunocompetency [10,19,20]. In individuals with normal immune function, adenoviruses cause restricted focal infections, the location depending on the transmission route. There is variation in the tissue tropism between different adenovirus serotypes, and adenoviruses can theoretically replicate locally in several different organs [20]. Disseminated adenovirus infections are mainly seen in immunocompromised patients and are usually connected with other severe systemic symptoms [24].

### 2.2. Adenovirus Infection

The adenovirus infection can be mediated through a variety of receptors. Some adenovirus serotypes infect host cells by attaching to the coxsackie-adenovirus receptors (CARs) with their fiber knobs. The CARs are present in the tight junctions between epithelial cells in many organs. This primary interaction is followed by the binding of the pentons to the α_v_β_3_ and α_v_β_5_ integrins on a cell surface, which initiates the internalization of the virus via clathrin-dependent endocytosis. There are also serotypes that bind to the membrane proteins belonging to the cluster of the differentiation system and some other surface structures [20,24].

**Figure 2:**
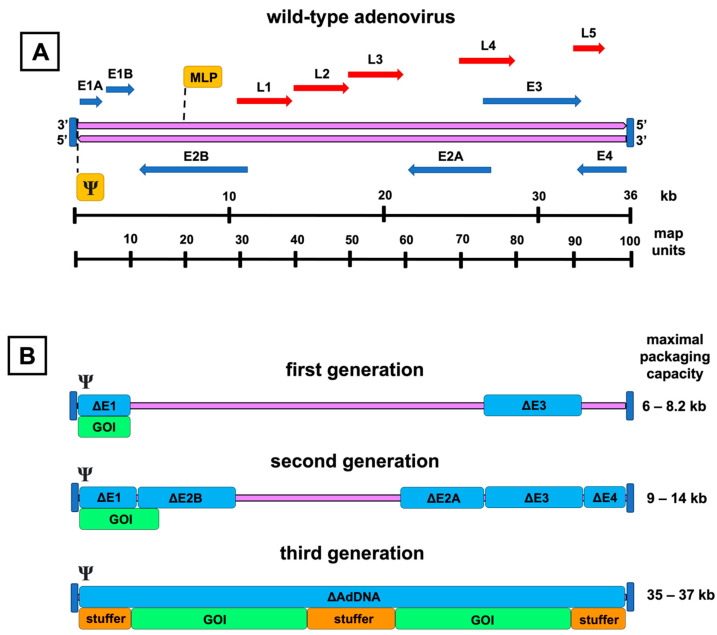
Genomic organization of the adenovirus and its vector derivatives. A schematic representation of the wild-type adenovirus genome and its transcription units is shown on top (**A**). The double-stranded adenoviral DNA (purple arrows) is flanked by inverted terminal repeats (vertical blue lines), and the packaging signal (Ψ) is at the left end of the genome. Early genes (E1–E4) are presented as blue arrows, and late genes (L1–L5) as red arrows with their respective transcription directions. Major late promoter (MLP) directs the transcription of the late genes. The picture below (**B**) presents the adenoviral vector generations with their respective genomic deletions (Δ), highlighted by the blue color over the adenovirus DNA (purple line). Under the deletion map, insertion spaces for the gene of interest (GOI) are shown. Inverted terminal repeats and packaging signals are present in each generation. Third-generation vectors are devoid of all coding adenoviral DNA (AdDNA). Additional stuffer DNA is inserted to reach the genomic size required for the encapsidation of the vector. The range for the maximal transgene packaging capacity (kb) is presented on the right.

Cytolysis, which takes place at the end of the viral replication cycle, and the subsequent virus release from the host cell are thought to be the result of the viral hijacking of the host translational machinery and accumulation of mature viruses inside the host cell. The proteins encoded by the regions E1, E2, and E4 enable adenovirus replication in the host cell (Figure 2A). In addition, adenovirus death protein (ADP) encoded by the E3 region plays a crucial role in cytolysis, and its overexpression increases viral spread into adjacent cells [25].

The tropism of adenoviruses varies greatly depending on the adenovirus species. Viruses from species A, F, and G infect gastrointestinal epithelial cells. Species C, E, and part of species B have tropism to the respiratory tract, and the rest of species B to the urinary tract. In contrast, adenovirus species D infects the conjunctival surface of the eye. HAdV-5 belongs to the species C and has thus natural tropism to the respiratory epithelial cells, but also to the hepatocytes [20].

Intravenous administration of wild-type HAdV-5 at a dose of 2 × 10^12^ vp results in biodistribution mainly in the lungs and liver in a porcine model. In addition, minor biodistribution occurs in the kidney, heart, skeletal muscle, brain, and gonads, but the concentration of the viral DNA is many thousand-fold lower than in the lungs [26]. Using immunohistochemical and hybridization-based assays, it has been shown that infection by certain adenovirus serotypes can remain latent in the tonsils, adenoids, and lungs [27].

Wild-type adenovirus does not integrate into the host genome in replication-permissive cells, and the infection of the host cell leads to its inevitable death by cytolysis at the end of the adenoviral replication cycle [28]. Some adenovirus serotypes have been found to be oncogenic in rodent cells [29]. Adenovirus serotypes coexisting in the same individual can recombine and form hybrids causing new infections, especially in immunocompromised but also in healthy individuals [30,31].

### 2.3. Environmental Aspects

Adenoviral shedding from an infected person takes place via respiratory aerosols and feces. Horizontal transmission can occur, for example, if viruses enter the respiratory system or the conjunctiva via droplets. Transmission by the fecal-oral route is also possible, especially in children [20].

Adenoviruses of serotypes 5, 8, and 19 can survive for up to 49 days on environmental plastic and metal surfaces [32]. A study comparing twenty-one disinfectants concluded that mixtures containing at least 1900 ppm of chlorine, 65% ethanol, and 0.63% quaternary ammonium compounds or 79% ethanol and 0.1% quaternary ammonium compounds should be used to disinfect surfaces contaminated with serotype 8 adenoviruses [33].

Although the efficacy of some antiviral drugs that alter the function of DNA polymerase has already been tested in clinical trials, no antiviral drugs for adenoviral infections have yet been approved for clinical use [24]. On the other hand, enteric vaccines based on replication-competent wild-type adenoviruses have been used to prevent acute respiratory distress syndrome in some risk groups, for example, in military personnel. These vaccines have been proven to be safe and well-tolerated [34]. After administering an adenovirus vaccine for serotype 4, fecal shedding of adenovirus takes place for 2 to 3 weeks, but the transmission to unvaccinated individuals using the same facilities, such as toilets, has not been shown to take place [27,35].

## 3. Adenovirus Vectors

Based on the extent of their genetic modifications, the replication-deficient AdVs are categorized as first-, second-, or third-generation constructs. Additionally, conditionally replicating or oncolytic AdVs form another distinct and important AdV group. These are described briefly in a later section as not being the primary focus of this review. Concerning the described genomic modifications and their roles, the schematic map of the adenovirus genome is shown in Figure 2A.

### 3.1. First-Generation Adenovirus Vectors

First-generation AdVs are the simplest of all adenoviral constructs. They contain a genomic deletion of the E1 region and an expression cassette of the preferred transgene (Figure 2B). The E1 region consists of two separate E1A and E1B transcription units. These units encode proteins to bring the infected cell into the state that allows viral particles to be produced and evasion of the virus from the host immune response activated by the synthesis of adenovirus DNA.

Two of the most abundant proteins encoded by the E1A unit are E1A 12S and 13S. The proteins are identical in their amino acid sequences apart from a 46 amino acids long segment in the middle of the polypeptide 13S. 12S and 13S regulate viral replication by binding to multiple cellular proteins, thereby shifting the cell cycle from the G_0_ phase to the S phase [19]. The E1B unit mainly produces two proteins called E1B 19K and E1B 55K. E1B 55K inhibits the tumor protein p53-induced apoptosis and increases the degradation of p53 as a complex with the E4 open reading frame (ORF) 6 protein [36,37]. E1B 19K also contributes to the inhibition of apoptosis by preventing the induction of tumor necrosis factor (TNF) α and Fas-mediated cell death pathways [19]. E1-deleted AdVs cannot replicate in human cells because the genomic region encodes transcription factors essential for adenovirus replication. If some vectors regain the deleted E1 region by recombination, they can replicate nearly as efficiently as the wild-type adenoviruses [13].

In addition to the replication enabling E1 region, the E3 region can also be deleted to produce more space for genomic insertions. Although not necessary for viral replication, the seven proteins encoded by the E3 region contribute to the protection of the virus from the host immune responses, apart from E3 12.5K, whose function is still unknown, and ADP, which serves as a factor to aid the cytolysis and release of the mature virions from the cell during the late infection [38,39]. From the rest of the proteins, E3 gp19K prevents the introduction of antigens by a major histocompatibility complex 1 allowing the infected cell to escape from the cytotoxic T cells [40], E3 receptor internalization and degradation (RID) α and β proteins form a complex on the membrane of the infected cell that inhibits TNFα and Fas-mediated apoptosis [41], E3 6.7K forms a complex with RID β, inhibiting the apoptotic cascade induced by TNF-related apoptosis-inducing ligand [42], and E3 14.7K participates in the inhibition of apoptosis caused by TNFα and Fas [43,44].

### 3.2. Second-Generation Adenovirus Vectors

Second-generation AdVs have the genomic deletions of the first-generation AdVs and additional genomic deletions in the regions E2 or E4, intending to reduce immunogenicity and increase the transgene packaging capacity even further (Figure 2B). The three translation products of the E2 region, divided into the promoter-proximal region E2A and the distal E2B, participate in DNA replication and are denoted as DNA-binding protein, precursor terminal protein, and adenoviral DNA polymerase [45]. The E4 region encodes at least seven different ORFs. These proteins have diverse functions, ranging from transcriptional regulation to the augmentation of late protein synthesis [46,47].

Despite the elegant rationale behind the additional modifications of second-generation AdVs, it seems that in terms of immunogenicity and toxicity, the deletion of the regions E2 and E4 offers only a limited benefit compared to omitting only the E1 and E3 regions [48,49,50]. Moreover, the transgene expression seems to be unstable [50]. In fact, at least one study reported that E4 deletion in the first-generation AdV enhances endothelial apoptosis compared to the control vectors [51]. On top of having no apparent benefit over the first-generation AdVs concerning safety and efficacy, the production yields of the second-generation vectors remain lower [52].

### 3.3. Third-Generation Adenovirus Vectors

Finally, the third-generation AdVs, also called gutless, gutted, helper-dependent, or high-capacity AdVs, have been modified to be devoid of all adenovirus DNA, aside from the sequences mandatory for the packaging of the expression cassette (Figure 2B). This also includes the late-phase transcription regions L1–L5, which are all controlled by the same major late promoter and encode proteins forming the capsid and participating in the assembly, genomic packaging, and regulation of the maturing virions [53].

Third-generation AdVs are attractive vectors for gene therapy. Due to up to 35–37 kB of removed adenoviral DNA, they have room for a larger transgene cassette and, at least in theory, are also less immunogenic and capable of more extended transgene expression [54]. Compared to the first- and second-generation, the production line of the third-generation AdVs is more complex and, as the name “helper-dependent” implies, requires a separate helper virus to provide all the necessary proteins for vector propagation [52]. However, the additional effort might be worthwhile, as the current evidence supports the capability of the third-generation AdVs to produce substantially longer-lasting transgene expression than the first-generation AdVs with less inflammation [55,56,57].

## 4. Vector Manufacturing

### 4.1. Construction Methods

#### 4.1.1. In Vitro Ligation

**Figure 3:**
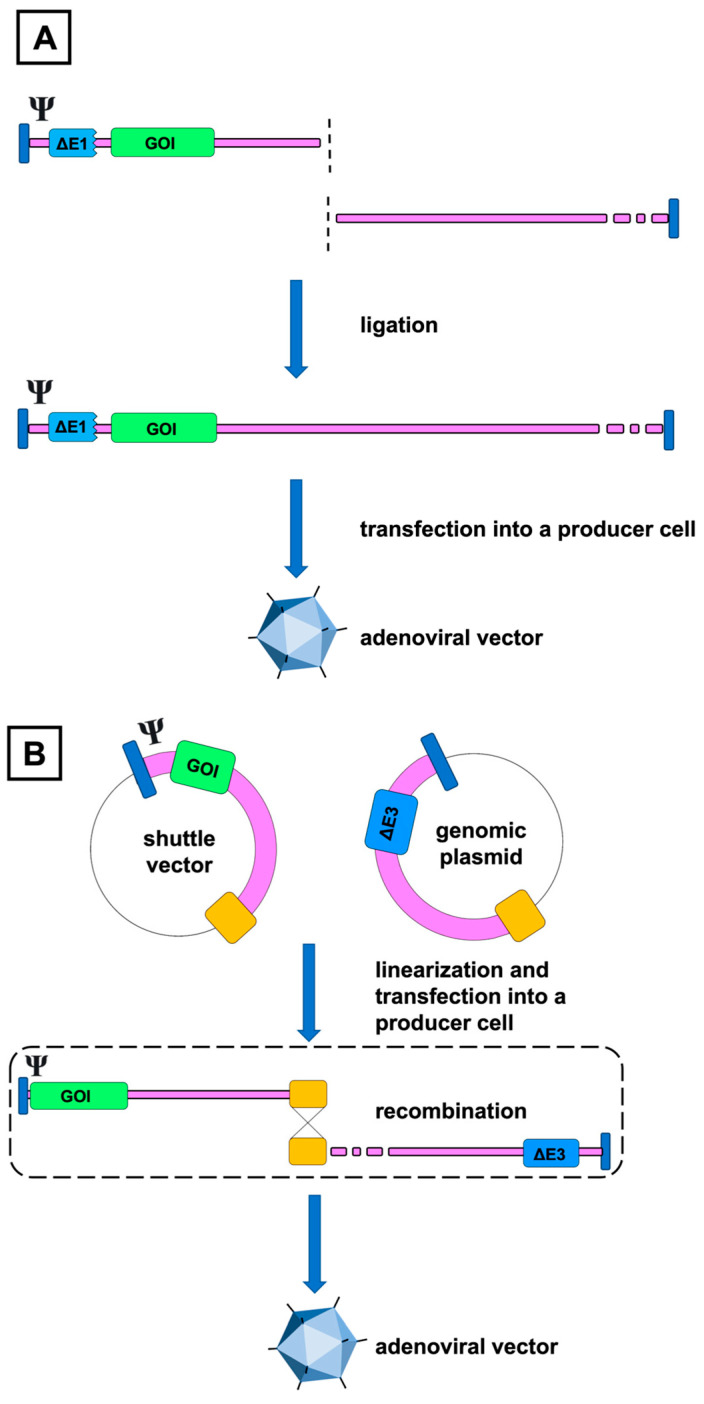
Simplified illustration of the two traditional methods for adenoviral vector production. In in vitro ligation (**A**), the restriction enzyme excised the left-end terminus with a desired gene of interest (GOI), E1 deletion, packaging signal (Ψ), and the left inverted terminal repeat, is ligated with the right-end part of the adenoviral DNA. In the early versions, localization of the available cleavage sites caused parts of the E1-region to be retained in the vector, limiting the space for the transgene. In the homologous recombination method (**B**), overlapping sequences cause homologous recombination of both genomic fragments to take place when transfected inside the producer cell, such as HEK293. The example presents the production of a first-generation vector using a genomic plasmid with right-end adenoviral DNA and an E3 deletion; however, predigested linear adenoviral DNA has also been used.

The two traditional methods for creating recombinant adenoviral DNA are based either on in vitro ligation or homologous recombination. In the first approach, the modified fragment from the left end of the adenoviral genome is ligated with the restriction enzyme digested adenoviral genome in vitro, resulting in the replication of a new adenovirus variant when the helper cells are transfected by the ligation product (Figure 3A) [58,59].

Although revolutionary at the time of its invention and proving that the adenovirus genome can be modified and propagated for practical uses, in its earliest form, the ligation method had major shortcomings in utility for large-scale AdV manufacturing. Due to the localization of the available restriction sites, most commonly *XbaI* for HAdV-5 dl309 mutant or *ClaI* for wild-type HAdV-5, most of the E1 region remained in the cleaved DNA, restricting the space for the transgene. Because of the small size difference between the naïve and digested adenoviral genomes, it was also hard to verify if the preliminary digestion process was complete. This posed a risk for contamination by non-cleaved DNA and subsequent enrichment of non-recombinant viruses due to their more efficient production. In addition, it was possible that the cleaved left-end fragments self-religated unintentionally, forming another source of contamination [60].

The method was later improved by introducing additional unique restriction sites, I-*Ceu*I, *Swa*I, and PI-*Sce*I, which corresponded more closely to the E1 region and addressed the problem related to the contamination by the parental virus [61,62,63]. Nevertheless, homologous recombination, discussed in the next section, has remained a far more popular strategy for constructing AdVs [64].

#### 4.1.2. Homologous Recombination

The second and, in its fundamental principle, still a widely used technique for constructing AdVs is based on homologous recombination. In this method, the E1-complementing HEK293 ([65]) helper cell is cotransfected with a shuttle plasmid containing an E1-deleted left end of the adenoviral genome and a transgene, and either a restriction enzyme-cleaved linear DNA or, more preferably, a second plasmid including most of the adenoviral genome but lacking the E1 region and the packaging signals. The overlapping sequences at the end of both DNAs enable homologous recombination, and the desired AdV DNA is formed intracellularly in the producer cell (Figure 3B) [66]. As neither of these substrates can generate mature adenoviruses independently, at least in theory, the resulting virions should be the outcome of their homologous recombination [66,67].

**Figure 4:**
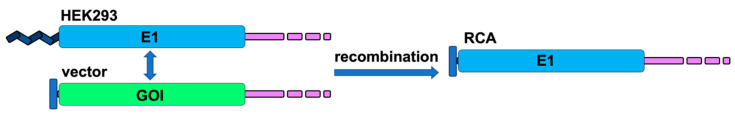
Homologous recombination between HEK293 cells and the adenoviral vector. Due to the marked sequence overlap between the production cell and the propagated vector, replication-enabling early genomic region is frequently gained back by the E1-deleted vectors. This results in adenovirus mutants which have gained back the E1 region but have lost the transgene in exchange.

The major drawbacks of the traditional homologous recombination technique are the low frequency of spontaneous recombination events as well as the high rate of RCA generation due to the secondary and undesired recombination between the AdV and the E1 region in the HEK293 genome (Figure 4), the latter eliciting the need for time-consuming plaque purification [59]. Furthermore, if restriction enzyme-digested right-end adenoviral DNA is used, the risk of parenteral virus contamination, described in the previous section, also applies to this method.

Strategies for improving the homologous recombination method involve incorporating *E. coli* for rescuing recombinant AdVs. Their benefits include a higher yield of the recombinant DNA due to efficient homologous recombination and cloning machinery in bacterial cells, which renders the tedious plaque purification required in the mammalian cell systems for generating seed stocks unnecessary [68,69]. Yeast can also be utilized as a platform for a more effective generation of recombinant AdV DNA [70,71]. Finally, although technically not based anymore on homologous recombination, the yield of rescued AdV in mammalian cell systems can be improved by inserting a gene sequence encoding Cre recombinase to the backbone of the adenoviral genomic plasmid which is left out from the recombinant AdV. When active, Cre recombinase drives the recombination of the loxP-flanked transgene expression cassette in the shuttle plasmid and adenoviral DNA in the genomic plasmid resulting in an enhanced rate of recombination [72]. For the third-generation AdVs, the Cre-loxP system is also used to excise the packaging signal from the helper virus and thus reduce the contamination by its progeny [73].

### 4.2. Vector Propagation

After the desired AdV candidate has been isolated with one of the previously described methods, the production continues with vector propagation and generation of virus seed stocks using cells that can provide the missing functions required for the replication in trans, such as HEK293 [52,60]. Despite being developed already in 1977, HEK293 has remained the most popular cell line for AdV development, although many other options exist, reviewed thoroughly by Kovesdi and Hedley [52]. In the case of the third-generation AdVs, the propagation is more complicated, and coinfection with a helper virus is needed to provide the gutless vector with the rest of its deleted DNA to facilitate the replication and packaging of mature virions [74]. In addition, a method for controlling the simultaneous amplification of the helper virus is required, such as the previously described Cre-loxP [73].

Once the genomic integrity of the newly prepared seed stock has been confirmed by electrophoresis, it can be used to inoculate larger cell cultures to produce high-titer lots [60]. After the incubation, the infected cells are freeze-thawed, and the resulting lysate is separated, for example, by cesium chloride gradient ultracentrifugation [75]. Finally, the product is characterized and purified from contaminants. In principle, industrial large-scale vector manufacturing follows these same steps. However, the process has been developed further to meet the strict regulatory standards while still being cost and time-effective [76]. Today, AdV propagation utilizes suspension bioreactors to produce batches that can easily meet commercial demands and supply late-phase clinical trials [77,78].

### 4.3. Formation of RCA

It has been consistently shown that the first-generation AdVs are prone to gain back their ability to replicate during repeated propagation passages in HEK293 cells due to homologous recombination between the vector and the producer cell (Figure 4), which is the predominant source of RCA in the production lots [13,79,80,81]. Further emergence of RCA can occur if these mutants augment the replication-incompetent vectors with the E1 functions by the mechanism of trans-complementation [15], in which case the transgene expression might also be enhanced [82].

The problem of RCA relates mainly to the first-generation AdVs, as the later-generation vectors also possess other genomic deletions, and hence regaining these regions is also required to transform the recombinant AdV to RCA [12]. The generation of RCA due to recombination between the E1 deleted helper virus and the producer cell is a notable exception to this and forms a commonly observed RCA source in the systems designed for producing third-generation AdVs [73].

**Figure 5:**
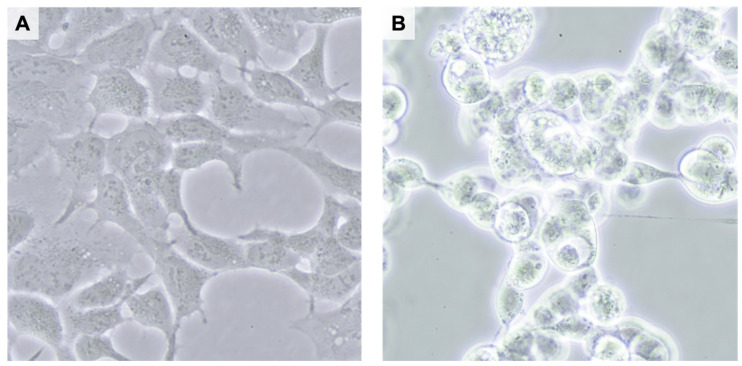
Cytopathic effect in HEK293 cells. The left picture shows the normal morphology of a viable HEK293 cell line in a high-density culture under light microscopy with 400-fold magnification (**A**). The right picture shows the cytopathic effect with the characteristic morphological changes in lytic adenovirus infection, including swelling and clumping of affected HEK293 cells and formation of inclusion bodies (**B**).

Traditionally, the assays for detecting RCA have been based on presenting a cytopathic effect (CPE) (Figure 5) in cells permissive to adenovirus replication, such as A549. In these assays, the tested substance is inoculated on the detector cells. After sufficient incubation, the cells are screened microscopically for CPE, indicating the presence of RCA [83]. In the tests provided by the commercial laboratories, the inoculated amount of vectors is usually the same as the FDA’s current upper limit for RCA, i.e., 1 RCA in 3 × 10^10^ tested vp [17].

Although the FDA’s current recommendation favors the use of CPE-based assays [16], these have been criticized due to their subjective nature and tediousness [18,84]. In addition, CPE testing requires a high detector cell per vp ratio because also the replication-deficient AdVs are cytotoxic to the detector cells in excess amounts and may thus restrict the replication of RCA [84]. The proteins encoded by the AdV may also interfere with the functions of the used assay cells [18].

As a result, more sophisticated and rapid PCR-based RCA assays have been developed [18,84,85,86]. In contrast to the tests relying solely on the visual observation of CPE, these assays either use quantitative PCR (qPCR) or droplet digital PCR (ddPCR) with probes targeted to the replication-specific E1 region to quantify the RCA released after one or more infectious passages in detector cells. The detector cells are needed to maximize the yield of RCA, minimize any potential interference from the protein encoded by the transgene [18], separate the RCA-derived E1 sequences from those originating from HEK293 residues, and confirm whether the detected RCA is infectious [85].

Recently, the development of primer specificity has also allowed a direct qPCR-based measurement of RCA contamination in oncolytic AdV batches without the need for amplification of the tested substance in helper cells [86]. The RCA testing methodology for replication-deficient AdVs would likely benefit from similar progress.

### 4.4. RCA Prevention

As the main reason for the emergence of RCA is homologous recombination in production cells, the primary method for preventing the formation of RCA includes using cell lines that minimize sequence homology with the E1 deleted AdVs. The best-known example is PER.C6, derived from human embryonic retinoblasts, which has been shown to produce similar yields of first-generation AdVs to HEK293 cells, but with minimal risk of the emergence of RCA due to recombination [80]. Although some consider PER.C6 the most suitable cell line for first-generation AdV production, their downside includes the restricted availability for manufacturers with a licensing fee and lesser adaptability for large-scale production [52]. Hence, HEK293 remains widely used in AdV development. In addition, even though occurring rarely, a small 177 nucleotide-long homologous fragment present both in PER.C6 and amplified AdV makes the E1 recombination still possible. However, the resulting mutant is replication-deficient due to deletions compensating for the large insertion from PER.C6 [87].

In addition to PER.C6, other cell lines that have been developed to produce first-generation AdVs with a similar rationale include HeLa-E1 [88], N52.E6 [89], and UR cells [90], transformed from cervical cancer cells, primary human amniocytes, and human embryonic lung cells, respectively. Owing to its tumorigenicity, however, HeLa-E1 is not allowed for commercial use [52]. For the producer cells of the third-generation AdVs, any present homology between the helper virus must also be considered, as it forms a significant source of RCA in these systems [91].

Another strategy to prevent RCA occurrence is to include additional modifications in the AdV genome rather than in the producer cell DNA. For example, omitting or relocating the sequence encoding pIX has been shown to reduce significantly, albeit not removing completely, RCA generation in HEK293-based propagation of the first-generation AdVs, because it comprises 50–60% of the homology between the AdV and the producer cell [79,81]. With these constructs, the pIX missing functions must be provided at a sufficient level in trans by the producer cell because it is required for delivering the virion its normal structural and thermal stability [52,92,93]. Furthermore, because the homology is not eliminated with this approach, the attempts to secure the absence of RCA still require producer cell engineering [81].

The systems designed to produce second-generation AdVs can probably be seen to be reasonably resistant to generating RCA, at least in forms in which all the deleted sequences would be regained, because it would require multiple recombination events to take place for causing the AdV to be capable of replicating functionally [12,94,95]. Whether the AdV mutants which are E1 positive but defective in other early gene regions, also called AdV revertants, could be harmful to the host is discussed in the next section.

Regarding the third-generation AdVs, there is no homology with the producer cell. Still, the helper virus has remained a problem since it is the substrate for the formation of recombinant RCA and residual contamination. To overcome this issue, Lee and colleagues have recently presented an alternative production method utilizing a helper plasmid that cannot be packaged into mature virions, excluding efficiently both the emergence of helper virus-originating RCA and the contamination by the helper virus itself [96].

## 5. RCA and the Safety of Gene Therapy

### 5.1. Preclinical Data

The first suspicions about the harmful effects of RCA arose from the preclinical studies, which showed that RCA induces a dose-dependent immunogenic reaction in batches contaminated with it. In their pioneering study in 1994, Lochmüller et al. found that RCA-positive β-galactosidase encoding first-generation AdV preparation manifested poor transduction efficacy and provoked an endomysial mononuclear inflammatory reaction and muscle fiber atrophy in the treated tibialis anterior muscle of the newborn rats. Similar reactions were not observed when using a preparation without a positive hybridization signal with the E1 probe [13]. Hermens et al. observed that the concomitant administration of RCA elicited degeneration of astroglial cells and diminishment of the transgene expression when injected into the parenchyma of the facial motor nucleus in immunocompetent Wistar rats but not in athymic nude rats, suggesting that the host response to RCA is mediated mainly by T-cells [14].

There is also evidence that the simultaneous presence of RCA delays the vector clearance and prolongs its persistence in the host by providing the missing E1-functions in trans, as shown by Imler et al. in their study of replication-deficient AdVs aimed at the treatment of cystic fibrosis with concurrent wild-type adenovirus infection in the targeted airway epithelia [15]. Another issue is that co-administered RCA can also result in increased transgene expression far beyond the levels achieved by the use of replication-deficient AdVs, which can be even desirable in certain specific contexts, such as when aiming to enhance the transgene expression in cancer cells otherwise poorly permissive to the adenoviral infection [97]. In the treatment of non-malignant diseases, however, the exact mechanism might expose the treated patient to transgene-specific adverse effects.

With regard to the revertant AdVs, caution has been raised about their possible oncogenicity considering that the genomic region E1 has the potential to transform and immortalize rodent cells and also some of human origin in vitro [12,98]. Based on their success in transforming baby-rat kidney cells using either E2 or E4 deleted but E1-positive AdVs, Fallaux et al. speculated whether revertant AdVs might be able to induce tumorigenesis in humans [12]. However, they also pointed out that since the E1 region encodes proteins that function as epitopes for cytotoxic T cells, this risk might be purely theoretical, at least in immunocompetent patients.

Whereas a sole deletion in the E4 region renders the AdV revertant replication deficient, an RCA having an E3 deletion in an otherwise intact genome is not only able to replicate similarly to the wild-type virus but its pathogenicity is actually increased significantly as well, as demonstrated by Ginsberg et al. in their study investigating the role of the E3 region using a cotton rat model for pneumonia [99]. This is especially problematic because, as shown by Lochmüller et al., the generation of RCA in HEK293 results in mutants that still carry the E3 deletion [13].

### 5.2. RCA in Clinical Trials

Due to the previously described safety concerns, it has been uniformly accepted that AdV products for clinical gene therapy trials should be as RCA-free as possible. Clinical data regarding the safety of residual RCA in the E1 deleted adenoviral investigational medicinal products (IMPs) have not been very relevant so far because it has been taken for granted that the industry and academic researchers using HEK293 cells for AdV propagation have been able to comply with the FDA’s requirement, i.e., upper limit of 1 RCA in 3 × 10^10^ tested vp.

Recently, this topic has gained more attention, as the CPE assays have become sensitive enough to detect traces of RCA from batches tested previously negative with the CPE methods. In fact, according to some estimates, the sensitivity of the old industry standard CPE assay, the so-called roller bottle assay, is far from the current upper limit, reaching only 75 RCA in 3 × 10^10^ tested vp [18]. For us, the ambiguity of the term ‘replication deficiency’ in relation to the first-generation AdV products became evident when we retested the clinical batches of the already completed Kuopio Angiogenesis Trial 301 (KAT301) with the more sensitive ddPCR assay.

### 5.3. Observations from Cardiovascular Studies

To our knowledge, KAT301 is the only clinical trial in which the IMP, HEK293-propagated replication-deficient HAdV-5 encoding for vascular endothelial growth factor-D^ΔNΔC^ (AdVEGF-D) [100], has been confirmed to be RCA positive using ddPCR after the completion of the trial, despite the initial negative test result [18]. According to the tests conducted on the batches from the same master virus seed stock, the range of RCA in the final phase 1 drug product was retrospectively estimated to be significantly higher than approved by the FDA, 100–200 RCA in 3 × 10^10^ tested vp [101].

**Figure 6:**
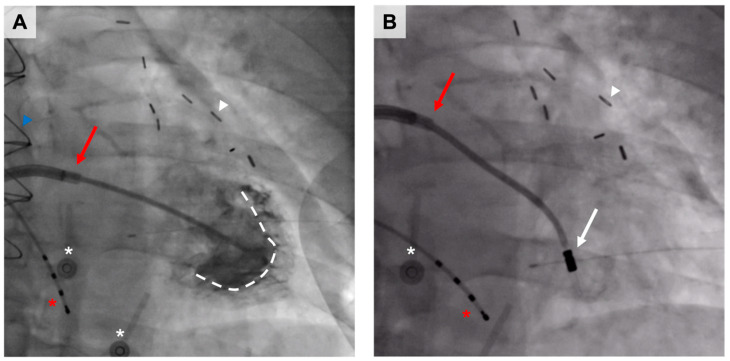
Intramyocardial gene therapy in a patient treated previously with coronary artery bypass grafting. On the left (**A**), the inner margins of the left ventricle (dashed white line) are projected by contrast media in the fluoroscopy using a transseptal guiding catheter (red arrow) in a right anterior oblique view. On the right (**B**), the intramyocardial injection catheter (white arrow) is in the vicinity of the target area, introduced in the left ventricle through the mitral valve. The other radiopaque elements related to the procedure are the temporary right ventricular pacing catheter (red asterisk) and the superficial electrocardiography electrodes (white asterisk). Sternotomy wires (blue arrowhead) and mediastinal clips (white arrowhead) are a remnant of past coronary artery bypass grafting.

In KAT301, 30 coronary artery disease patients suffering from chest pain despite optimal medical therapy and who were not eligible for interventional coronary procedures were randomized to receive either intramyocardially administered AdVEGF-D or placebo (Figure 6) [100]. The encoded transgene, VEGF-D^ΔNΔC^, belongs to the larger VEGF family and is a proteolytically processed form of its precursor, full-length VEGF-D, exerting its functions through two tyrosine kinase receptors, VEGF receptor (VEGFR)-2 and -3 [102,103]. Preclinical studies conducted before KAT301 have shown that AdVEGF-D inflicts potent angiogenesis in porcine myocardium [104,105,106]. In addition, AdVEGF-D also induces arteriogenesis and, given its affinity to VEGFR3, is also a lymphangiogenic growth factor [107].

No adverse reactions fulfilled the criteria for a suspected unexpected serious adverse reaction or raised any concern for the presence of RCA. Transient fever, the elevation of C-reactive protein, and thrombocytopenia were observed in the patients treated with AdVEGF-D. The changes were mild and resolved without further sequelae. An excellent safety profile was also observed in the long-term follow-up, with no significant differences in the incidence of major adverse cardiovascular events, comorbidities including cancer, or arrhythmias between the groups [108,109].

Although any conclusions about the safety of the residual RCA drawn from our clinical trials before KAT301 remain speculative as no post hoc RCA testing with the more sensitive methods has been conducted, the earlier clinical studies using angiogenic first-generation AdVs have likely used batches with comparable amounts of RCA because the manufacturing methods have been similar. Our group’s previous angiogenic gene therapy studies have neither witnessed any adverse effects that could have raised any concerns about the possible role of RCA [110,111,112]. Correspondently, in our past trials, the safety profile of similarly manufactured and supposedly replication-deficient, thymidine kinase-encoding first-generation AdVs designed for treating malignant glioma has been acceptable [113,114]. We plan to include probing toward E1 in our future gene therapy trials using the first-generation AdVs to provide more robust data on the subject.

### 5.4. Safety Testing

Concerned that our angiogenic gene therapy IMPs are not, strictly speaking, RCA-free, we wanted to elucidate the effects of residual RCA at the levels formed during Good Manufacturing Practice-compliant manufacturing. In a preclinical safety study, we compared three clinical-grade AdVEGF-D production lots graded with different RCA content (<10, 10–100, and 100–200 in 3 × 10^10^ tested vp) in a rabbit hindlimb model for their angiogenic potential, toxicity, and vector biodistribution and shedding (Table 1) [101].

Contrary to the previous preclinical studies indicating the potential RCA-related hazards, we were surprised to find that the RCA level was not associated with any changes in the tested safety or efficacy parameters. Histologically, the gene therapy resulted in an accumulation of mononuclear inflammatory cells in the injected semimembranosus muscles in each group. However, these changes were subjectively more prominent only in the group with the highest RCA level. Most strikingly, we could not detect RCA-specific E1-sequence in any of the tested biological samples, including the transduced hindlimbs. However, despite these reassuring results, it needs to be mentioned that even at their highest, the compared levels were far below those used in the earlier preclinical studies, for example, by Fechner et al. demonstrating an enhancer effect when coinfecting cancer cells with marker gene AdV and RCA at a 240:1 ratio [97].

### 5.5. Oncolytic Adenoviruses

As the clinical safety data regarding residual RCA are almost non-existent, another valuable source of evidence comes from studies investigating restrictively replication-competent or oncolytic adenoviruses. Oncolytic vectors are unique in that they are designed to replicate in a controlled manner in cancer cells, causing tumor lysis. ONYX-15, for example, is an adenoviral construct bearing an intact replication-permissive E1A region without the p53-suppressive E1B region, allowing replication theoretically only in cancer cells lacking the p53 function [115]. Due to the reports of the non-specificity of the replication with the earlier constructs [116], additional efforts have been made to improve the selectivity of oncolytic adenoviruses [117].

In a review article by Schenk-Braat et al., the shedding of oncolytic adenoviruses was investigated by examining 11 original studies [118]. In the studies included in the review, oncolytic adenoviruses were found to shed to blood from a few hours up to 76 h after their administration. There was no evidence of shedding to the skin. In two of the studies, it was possible to measure two different peak concentrations of adenoviral DNA from the blood, which was thought to be caused by adenoviral replication. Although infectious viral particles could not be detected from the blood, in one of the studies that investigated the use of oncolytic adenoviruses for prostate cancer, the presence of infectious viruses in urine was reported for up to 8 days after the injection into the prostate tumor. Historically, wild-type adenoviruses have been used to treat cervical cancer, with no clinical or pathological evidence of systemic virus disease [119].

## 6. Concluding Remarks

The finding that the clinical AdV stocks may contain higher amounts of RCA than earlier expected has revealed an important new aspect that should be considered as a part of the safety monitoring in future adenoviral gene therapy trials. The current preliminary data, fortunately, suggest that the traces of RCA inevitably formed during HEK293 propagation might not pose a significant safety risk as previously hypothesized. However, these cautionary conclusions have been derived only from the context of angiogenic gene therapy studies, and the situation might be different with AdVs armed with different transgenes and indicated to other pathologies, such as neurological diseases.

It is essential that all investigators are familiar with the topic of RCA and its potential risks since unpredictable adverse reactions might be possible in specific contexts, such as when treating immunocompromised patients or targeting tissues more susceptible to adenoviral replication. In the future, the safety characterization of RCA could be taken further by comparing a more comprehensive array of RCA levels in different preclinical models and including RCA-specific E1 probing in the routine blood sample assessments of the clinical trial recruits. Gene therapy will continue to change the paradigm of clinical medicine and offer tools for controlling previously untreatable conditions. For gene therapy investigators, gaining as much knowledge on this previously unaddressed topic is a way to respect the “do not harm” principle in medicine.

## Figures and Tables

**Table 1:** Setup of the preclinical RCA-AdVEGF-D safety study [101].

Animal Model	Female NZW Rabbit; a Dose of 1 × 10^11^ vp in Ten 100 μL Injections in the Non-Ischemic Hindlimb		
Group and administered preparation	AdVEGF-D low RCA	AdVEGF-D moderate RCA	AdVEGF-D high RCA
Sample size (*n*)	5	5	6
RCA level (per 3 × 10^10^ tested vp)	<10	10–100	100–200

Abbreviations: AdVEGF-D, adenoviral vascular endothelial growth factor-D^ΔNΔC^; NZW, New Zealand-white; RCA, replication-competent adenovirus; vp, viral particles.

## Data Availability

No new data were created or analyzed in this study. Data Sharing is not applicable to this article.
